# Anatomy and Physiology

**Published:** 1838-01

**Authors:** 


### PART THIRD.

### Selections from the Foreign Journals.

---

### ANATOMY AND PHYSIOLOGY.

### *On the Anatomy of the Eyelids.* By Dr. Zeis, of Dresden.

[It is not so much from the complexity or from the beautiful construction of the eyelids, that their anatomy is regarded with interest, as from their liability to many diseases, some of which are attended with great pain, and are even apt to prove fatal to vision. Our ophthalmological readers are well aware of the numerous and various structures of which the eyelids are composed. In the present article, Dr. Zeis has with great care reexamined several of these structures, and has communicated some new views concerning them, which we regard as important. We shall abridge Dr. Z.'s paper, and add a few comments of our own.]

1. *Skin.* The skin of the eyelids is very thin, destitute of hairs, and, according to Dr. Zeis, presents no trace of sebaceous follicles. Certainly it differs widely in this last respect from the skin of the nose, where the follicles are very evident; but we cannot suppose the skin of the eyelids to be destitute of sebaceous follicles, and we presume that the albuminous tumours, which are not unfrequently met with in this situation, are enlargements of these organs.

2. *Cilia.* We have already (vol. II. p. 454, and Plate II. fig. 2,) given an account of the structure of the hair-follicles and hair-glands, according to Gurlt. Dr. Zeis's researches on the cilia have led him to conclusions somewhat different.

The small whitish bodies, described by Dr. Zeis as hair-glands, lie nearly equidistant from the bulbs of the hairs and their exit through the skin, and surround each eyelash, he says, like a wreath. They are visible to the naked eye, but under the microscope they are seen to consist of a number of little grains. Eble, in his work entitled *Lehre von den Haaren*, represents these bodies, but speaks of them as merely particles of fat, and does not seem to regard them as peculiarly belonging to the hairs. Zeis contends that fat exists only under the skin, whereas these bodies are in the cutis, and within the hair follicles. That Eble has not been sufficiently attentive to them, is evident from his omitting them even when describing and figuring the whiskers of the lower animals, which he regards as the normal, or most perfect, hairs. The roots of the large whiskers on the snout of the ox are surrounded, according to Zeis, each by a very firm fibrous bag, filled with a reddish fluid, and including the hair bulb. If this bag or follicle is slit open in all its length, the little yellowish bodies are discovered surrounding the whiskers, exactly as they do the hairs on the body of the ox, which are not contained within any bag. A transverse section is given by Zeis, exhibiting the whisker, surrounded by a wreath of glands, the whole being contained within the fibrous follicle or hair-bag. The bristles near the eyebrow of the hog are also contained within such follicles, but Zeis could not detect any glands within the follicles in this animal.

The small hairs, which (with the exception of the palms and soles) cover every part of the human body, are destitute of the little glands, and their bulbs are placed merely in small mucous follicles, very similar to the sebaceous cryptæ. But the glands are found surrounding the hairs of the head, those of the beard, and the cilia. In man the glands are generally nearer to the surface, say about the exterior third or fourth of the bulb; but in the hairs of the body of the ox, generally in the middle between the bulb and the exit of the hair from the epidermis. From the regularity of their presence, as well as their being composd of little grains or acini, Zeis rejects the idea that these bodies are particles of fat, and considers them of a glandular nature. They may be destined either for the nourishment of the hairs, or for furnishing them with an oily varnish.

Gurlt represents these hair-glands, but with several material differences from the views of Zeis. The former represents the acini as much smaller than they appear to the latter. He asserts that in the neighbourhood of the root of each hair, there are generally two, rarely only one, gland. Had he made a section perpendicular to the shaft of the hair, Zeis thinks he would have immediately discovered his error upon this point, for the glands encircle the hair like a wreath. Zeis thinks Gurlt must have made his sections always in the diameter of the hair-follicle, that is, longitudinally. Zeis has not been able to perceive the duct which Gurlt figures going from each gland, and conducting the secretion of the gland into the cavity of the hair-follicle. Gurlt compares the hair-glands to the sebaceous glands, which he calls inversions of the skin. But whatever be their function, whether they contribute to the growth of the hair or serve merely to give it an oily varnish, it is plain they are of a much more complicated structure than the sebaceous follicles, even on the supposition, to which Zeis inclines, that they have no such slender duct as Gurlt represents. A most material point of difference between the representations of these two authors is, that Gurlt figures the glands as lying without, Zeis within the hair-capsule.

3. *Ligamenta Tarsea Lata.* Winslow described these ligaments as stretching from the edge of the orbit to that of the tarsi, with which he thought they formed a complete layer of the eyelids. Zinn could not trace the ligaments all the way to the tarsi. Haller doubted altogether of their ligamentous texture, and Zeis regards them as merely cellular substance.

4. *Meibomian Glands.* It is generally stated in anatomical works, that the acini, of which each row of the Meibomian glands is composed, communicate, so that their secretion passes from one to another, till it exudes on the edge of the eyelids. A very different idea was adopted by Morgagni, who maintained that each row of acini contained within it an excretory canal, along which the secretion of the whole acini is conveyed. This view is also that of Dr. Zeis, who establishes it by numerous observations both in the lower animals and in the human subject. He states that the canal in man is exceedingly narrow, and its parietes very thin. In brutes, its cavity is comparatively ample, especially in ruminants; the aperture by which it penetrates through the skin is always much contracted. He thinks that in man the canal must give off ramifications or side-branches, as the acini often lie apart from one another: and observes that the rows do not run close together, much less one heaped upon the other, as Soemmering represents them, but in separate lines, and even, in the same row, the acini generally distinct one from another.

The Meibomian glands are evidently conglomerate; and, notwithstanding Müller's assertion regarding the simplicity of their structure in the dog, in no animal are they mere follicles or inversions of the skin. In the dog, the acini are very minute; but still evident under a high magnifying power.* As they appear in the calf, Zeis compares these glands to the pancreas. They present, he says, in that animal, lobes, lobules, and acini. They are comparatively shorter in brutes than in man; and are generally of about the same length in the upper and lower eyelids; whereas in the human subject they are much longer in the upper than in the lower eyelid.

5. *Tarsi.* Anatomists tell us, that the Meibomian glands lie between the tarsi or cartilages of the eyelids, and the conjunctiva. Some say the glands are lodged in furrows, formed on the internal surface of the tarsi. If we look at the edge of the eyelids, it is plain that the apertures of the Meibomian canals are in the substance of the tarsus, and not between the conjunctiva and the cartilage. Dr. Zeis goes farther, however, than this. He maintains, that, in man, the Meibomian glands are entirely lodged in the substance of the tarsi; and that in very few of

* This we state on our own authority. The fact is not noticed by Zeis.—Rev.

the lower animals is there any cartilage at all in the eyelids. He says, that on making sections of the human eyelids, either perpendicular or transverse, it is evident that the Meibomian glands are on all sides surrounded by cartilage, and not nearer the posterior than they are to the anterior surface of the tarsus.

In the lower eyelid of the human subject, Dr. Zeis says, he has never been able to discover a true cartilaginous lamina. He thinks the Meibomian glands are lodged there, as they are in brutes, merely in dense cellular substance. In the upper eyelid of the sow, there is a cartilage; but there is none in the eyelids of any other of the lower animals. When we take the eyelid of a calf or of an ox between our fingers, the edge of the eyelid feels so firm and hard, that, without knowing the fact, we should never doubt of the existence of a tarsus. The eyelid of the horse, again, feels soft and relaxed. On dissection, it is manifest that the degree of firmness or of softness depends merely on the state of the cellular substance.

The question naturally arises, Why are the eyelids of man, and especially his upper eyelid, provided with so peculiar a part as the tarsus, of which the eyelids of brutes are deprived? Dr. Zeis is of opinion, that this contrivance is not so much for protecting the eye of man, as for allowing him the widest possible opening of the fissura palpebrarum. Were it not for the tarsi, whenever we opened our eyes, the fissura palpebrarum would become shortened from angle to angle, and we would thereby lose the advantage, which we at present possess, of seeing objects placed laterally, by a simple movement of the eyes from side to side. Brutes possess this lateral movement in a very imperfect degree; but for this they find a compensation in their neck, which is generally long and moveable. When their eyes are open, little more than their cornea is exposed, and very little of their sclerotica, so that a lateral movement of their eyes would hide their cornea behind the angles of their eyelids. Their fissura palpebrarum assumes, then, almost a circular form when their eyes are open. A tarsus would prevent this, as we may observe it to do in the sow. Man's compensation for the shortness and limited mobility of his neck is the tarsi, by means of which his fissura palpebrarum is elongated, his eye allowed to turn advantageously from side to side, and his lateral field of vision increased. *Ammon's Zeitschrift.* B. II. 3ter u. 4ter. Heft.

---

*Microscopical Researches on the ultimate Structure of the Nerves, and the central Parts of the Nervous System.* By Dr. BERRES, Professor of Anatomy at Vienna.

[CIRCUMSTANCES unnecessary to mention here have hitherto prevented us from laying before our readers an account of Ehrenberg's discoveries respecting the microscopical structure of the brain and nerves. The following abridgment of an important paper by Berres, contains an extension of some of Ehrenberg's views, which we hope soon to give at length.]

The forms in which the nervous substance presents itself to us under the magnifying glass, can only be compared to those of canals and vesicles; but, whether it be really hollow, and thus adapted for the reception of more delicate matter or fluid, or whether it does, in fact, contain fluids or an albuminous substance in a half-coagulated state, is still involved in great obscurity. In his numerous researches, Dr. B. only saw, in the larger canals and vesicles, when they were exposed to heat, phenomena strengthening the supposition that they were receptacles for delicate fluids, some of them, under these circumstances, expanded to double their usual diameter. Dr. B. only calls the structural elements of the nerve which are distinctly visible under a glass magnifying 750 times, canals and vesicles, because the shapes of the latter are those which they nearest resemble. The relative position and connexion of these elements, and the diameter of the canals and vesicles, are very diverse; and it is this diversity on which Dr. B. has founded a division of these structures into three natural classes, and of each class into several orders.

This new classification, if proved correct, will be of great value, inasmuch as it professes to indicate the *function* of nerves, the use of which is unknown, and expresses their analogy with others of the same class. All the nerves of the human body may be divided, as to their ultimate structure, according to Dr. B., into three classes: 1*st Class.* Nervous structures composed of canals swollen at intervals, like a string of beads, (*tubuli moniliformes.*) 2*d Class.* Ditto, composed of delicate canals, with vesicles attached, (*tubuli baccati.*) 3*d Class.* Ditto, of intussuscepted, invaginated canals, (*tubuli invaginati.*) Of the first class, are the nerves of the particular senses; of the second, the nerves of sensation; of the third, the nerves of motion. After a long description of his classes and orders, our author arrives at the important result, that in all the higher organs of sense, may be demonstrated, firstly, a nerve of a particular sense; secondly, a nerve of sensation; and, thirdly, one or more nerves of motion.

Other alleged discoveries of Dr. B. are the following: The structure of the brain and of the spinal marrow is similar to that of the first class of nerves. The nerves of a particular sensation and the sympathetic seem to run into the nervous centres without alteration or interruption; but those of the other classes lose their tegumentary membranes as they approach the latter, and change, thus, their structural arrangements, whilst the canals which compose them terminate by interlacing with the canals and vesicles proper to the brain; all these form together the higher organization of the nervous centres.

*Oest. Med. Jahrb.* Vol. ix.

---

*On the Microscopic Structure of the Brain, and of the Tissues intermixed with it.* By Dr. Luigi Calori.

After describing the three principal forms of the ultimate structure of the brain observed by anatomists, viz. the tubular of Ehrenberg, the fibrous or cylindrical of Alex. Monro and Fontana, and the globular of Della Torre, he mentions his own observations, which have led him to conclude that all these forms may be produced and rendered apparent by attention to certain particulars. The most uniform appearance is the globular; but the shape varies much with the mode of preparing the brain for examination, and with the degree of illumination to which it is subjected under the microscope, as well as with the magnifying power: nevertheless the globular is most probably the real form of the nervous matter.

The tissues that are intermingled with the nervous substance and give it support, are very fine and transparent cellular substance, and the blood-vessels pointed out by Prochaska, of which Calori asserts that no one has hitherto given a full and perfect description. That the vessels of the medullary and cortical portions are not identical is clear from their difference of caliber and distribution, and hence Gall was wrong in asserting that the latter was the source of the former. These vessels form a remarkably fine network, quite disproportionate to the size of the primary vessel, and similar to the distribution of the vessels on the surface of the Allantoïd membrane. The nervous matter is united to these vessels within whose ramifications it is collected; and, if really globular, it approaches most closely to the adipose tissue of Malpighi in its general appearance.

*Bullettino delle Scienze Med. de Bologna.* Sett. 1836.

---

*On the Fibrous Membrane beneath the Pleura Pulmonalis.* By M. Bazin.

After quoting the opinion of Colombo, the pupil and successor of Vesalius, that the pleura is composed of two laminæ, between which the blood-vessels and nerves are distributed, the author of this memoir states that the researches of succeeding anatomists have led to a different conclusion, in consequence of their having been confined to the pleura costalis. His own extended investigations into the structure of the respiratory organs in the series of vertebrated animals, have led him to the conclusion that the lungs, like other organs, possess a proper capsule. He remarks that capsular envelopes are found to exist in three states: 1, a fibrous network with large interstices, frequently taken for cellular tissue; 2, a complete fibrous or sclerous membrane, whose density and thickness may be variable; 3, an osseous plate. The capsule of the lungs in most animals presents the first condition, that which exists in man; but in the elephant, a distinct fibrous membrane is met with, consisting of bundles of parallel fibres interlacing at certain points with others, like the muscular coat of the bladder. The lung of a panther, which died of phthisis, presented M. Bazin with a hypertrophied condition of this membrane, which in the healthy state of this animal is not thicker than in man.

*Annales d'Anatomie et de Physiologie.* No. I. 1837.

---

*Examination of the Bodies embalmed by* Signor Tranchina.

In our Fourth Number (vol. II. p. 543,) we gave a short account of Tranchina's discovery of a method of preserving dead bodies by the injection of a solution or mixture of arsenic into the arteries. Two bodies were thus embalmed, three days after death, at the military hospital of Naples; and the following is the account of the Medical Faculty of the University appointed by his majesty to examine and report thereon.

The two bodies, injected on May 15, 1835, at the military hospital, and dissected on December 11, 1835, exhibited the following appearances:

The integuments were wrinkled and the flesh dried and shrivelled. The skin of one was livid-red, and blackish-red of the other. There was no mark of decomposition in either; they were only covered with mould.

The brain and cerebellum were in their natural state, firm and elastic, but diminished in size. The cerebral tissue was wonderfully preserved; the cortical substance alone had become brownish.

The heart was in its natural state. The lungs were somewhat dried and softened; but, when cut into, they gave no signs of putrefaction, nor any disagreeable smell.

The abdominal viscera were rather dry and flaccid, of a dark colour, but inodorous. The fæces and urine, in their respective receptacles, gave no proofs of putridity.

The arteries were empty, shriveled, and contained saline concretions. The veins contained liquid uncorrupted blood, that readily followed the incisions.

*Filiatre Sebezio. March,* 1836.

---

*On the Action of Strychnia on the Nervous System.* By Dr. H. Stannius.

In the following experiments, the nitrate of strychnia was preferred, on account of its greater solubility, to the other preparations of that alcaloid. Dr. Stannius introduced, beneath the integuments of strong healthy frogs, a few drops of a concentrated solution of the nitrate, and the following effects were generally observed: The animal continued to comport itself as usual for a short time, but, in from five to fifteen minutes, it became more quiet, taking at intervals short leaps, till one tremendous bound succeeded, followed instantly by the most energetic tetanic convulsions, which continued without interruption for about a minute. They then generally ceased; but the slightest shaking of the table, the least touch, or even the vibration of the air caused by speaking, sufficed to reproduce them instantly. This acute sensibility gradually diminished; which consequence Dr. Stannius ascribed to the absorption of a larger quantity of the poison. In analyzing these effects, we observe, firstly, an affection of the voluntary muscles; and, secondly, a greatly increased susceptibility to all external impressions. These phenomena may be owing, firstly, to the action of the poison on the brain; secondly, to its action on the extremities of the centripetal nerves, which, by reflecting it upon the brain and spinal marrow, might produce the anomalous phenomena; or, lastly, it may be owing to the direct action of the poison on the spinal marrow. The first supposition is negatived by experiments, which shew that the destruction of the brain does not prevent the occurrence of tetanic convulsions after application of the poison, and by others in which the spinal cord was divided above the origin of the nerves of the posterior extremities, and in which convulsions nevertheless ensued, both in the anterior and posterior half of the body. Secondly, does strychnia act primarily upon the extremities of the nerves through the instrumentality of the blood?

The following experiments may be regarded as answering this question in the negative: the spinal column of a frog was divided immediately above the origin of the nerves of the posterior extremities; the posterior portion of the column was then carefully separated from the soft parts, and the blood-vessels supplying it cut, so as to leave it in connexion with the other parts of the body solely through the nerves of the posterior extremities. These nerves were then freed from all adhesion to the adjacent vessels, muscles, &c. in that part of their course which lies between the spinal column and thighs; so that in this manner the posterior portion of the spinal marrow, and, to some extent, the nerves arising from it, were completely isolated from the blood-vessels. The blood-vessels and nerves retained their normal situation in the extremities, in which the circulation continued to be carried on, and mechanical irritation of the toes of the posterior extremities, by inducing the animal to retract them, shewed that the functions of the posterior half of the medulla continued to be performed. If the poison, therefore, act upon the extremities of the nerves, and its action be through them reflected upon the spinal cord, we should still expect tetanic convulsions both in the anterior and posterior half of the body. But this is not the case; the posterior extremities are not convulsed, although they still contract on the application of mechanical irritation. We thus arrive, by negative proof, at the conclusion that strychnia acts primarily upon the spinal marrow, and that from it result both the convulsions and increased excitability.

Here the question arises, Do these convulsions suppose also an alteration in the centripetal nerves, or is the excitability of the spinal marrow increased to such an extent that, without any change in these nerves, common impressions suffice to produce the most fearful spasmodic paroxysms? To decide this question the following experiments were instituted: the spinal canal of a frog was laid open, and the posterior roots of the nerves of the posterior extremities cut; the animal was then poisoned, and the usual phenomena appeared: the posterior extremities were fully convulsed, but mechanical irritation had no effect upon them, and the convulsions in the posterior extremities ceased as soon as the cord was divided above the origin of their nerves. Again: the spinal canal of a frog was laid open, and the posterior roots of the nerves of the posterior extremities cut across. The cord was then divided above the origin of these nerves, and the animal poisoned. No convulsions followed in the posterior extremities, nor did irritation of the spinal ends of the cut posterior roots produce them. These experiments were repeated several times with like results; and hence Dr. Stannius infers that the centripetal nerves are necessary for the appearance of tetanic convulsions, and that increased susceptibility to external impressions is not confined to the spinal marrow; as, in that case, irritation of the spinal ends of the cut posterior roots, or of the posterior aspect of the spinal cord itself, would give rise to the peculiar tetanic convulsions. We know, from the above experiments, that the poison is devoid of influence upon the extremities of the centripetal nerves, and we thus necessarily arrive at the conclusion that the centripetal nerves receive from the spinal cord an increase of their excitability; and that, thus charged, they react upon the medulla and occasion the peculiar convulsions.

In experiments with strychnia, the blood has generally been regarded as the vehicle of the poison. This opinion derives confirmation from the facts that some time is required after the introduction of the poison beneath the integument for its action to become apparent, and that no effect follows its immediate application to the spinal cord or nerves. Dr. Stannius was unable to recognize any alteration iu the appearance of the blood of poisoned frogs by means of the microscope, and he disproved the opinion that the act of respiration is necessary to impart to blood already charged with the poison, the power of acting upon the central organs of the nervous systems, by shewing that the same symptoms occurred in frogs which had previously been deprived of their lungs.

Professor Müller has lately called attention to the fact, that a solution of the aqueous extract of opium is possessed of a purely local action upon nerves with which it is brought in contact. To ascertain in how far strychnia possesses the same property, Dr. Stannius prepared three posterior extremities of frogs. The extremities of the nerves of one were steeped in a solution of opium; those of a second in a concentrated solution of strychnia; and those of the third in pure water. The nerves of the first shewed no susceptibility of mechanical irritation beyond a quarter of an hour; those of the second retained their excitability for an hour; and those of the third, for several hours. The stimulus of galvanism continued to affect the extremities of the nerves of the first for three-quarters of an hour; those of the second for an hour and a half; whilst those of the third, at the end of two hours and a half, still retained some excitability.

*Müller's Archiv.* Heft ii. 1837.

---

*Experiments on Animals with the Blood of Cholera Patients.*
By Dr. NAMIAS, of Venice.

UPON opening the body of a man, aged fifty, who had died in the cold stage of cholera after twenty-four hours' illness, at the Hospital of St. Daniel, the blood in the cavities of the heart was found to be black and congealed together, with one or two polypous concretions. A portion of the congealed blood, of the size of a strawberry, was inserted, without causing much suffering, under the skin of the thigh of an old and fat rabbit. The fur was shaved off, and an incision having been made through the skin, it was separated by the handle of the scalpel from the cellular tissue beneath; and into the cavity thus formed the blood was introduced, and the wound was then carefully closed with sutures. This mode of inoculation was followed in all the experiments.

*Experiment* 1. Five days after the operation the rabbit appeared ill, its evacuations were less solid than usual, and a whitish glutinous matter was observed upon the ground. The animal was found dead on the eighth day. The blood of the heart was black and grumous, with some fibrinous concretions; the bladder was full of urine; the injected blood had pervaded the surrounding tissues; the lips of the wound had their normal consistence; the internal surface of the whole of the skin was covered with blue spots of ecchymosis; but the rest of the organs exhibited no alteration.

*Experiment* 2. An equal quantity of the blood of this rabbit was injected in a similar manner into the thigh of a grey female rabbit. It was found dead in twenty-four hours afterwards, with the same appearance of the whitish matter upon the ground. The body when examined exhibited similar results as in the former experiment. The author here judiciously remarks that the rapid death of the second rabbit arose not only from its weaker age and sex, but also from the blood of an animal of the same species being more readily absorbed than human blood.

*Experiments* 3 and 4. The blood of the last rabbit was inserted into two others that died in six days with the same marks of disease. These rabbits were much larger than the former.

The next experiments were performed with the blood of a man who died in the cold stage after twelve hours' illness. The blood was black and grumous, but there were no concretions.

*Experiment* 5. This was a fat old grey rabbit of the male sex. It died five days after the operation. There was no whitish matter on the floor; the bladder was empty; there were many brown spots on the inner surface of the skin. The state of the blood and of the wound that was not of a kind to affect the health of the animal, was the same as in the other cases.

*Experiment* 6. Performed with the blood of the last on a male rabbit two pound and a quarter in weight. It died in forty hours. Bladder full; blood black and fluid; the usual spots on the skin. In this instance also, the poison transmitted through a similar animal acted more rapidly than when taken directly from man.

*Experiment* 7. Another rabbit, inoculated with the blood of the last, was found dead in six days.

*Experiments* 8 and 9. In these two experiments, for the sake of greater accuracy, the doctor weighed both the rabbits and the blood. Into one, weighing three pound and a half, he injected ten grains of blood from the last experiment; and into the other, weighing three pounds, he inserted eight grains of the same blood. Both died in six days.

The next experiments were undertaken with the view of ascertaining whether human blood taken from persons not dead of cholera would produce the like results. Three rabbits were inoculated: one, of two pounds and a quarter, with seven grains of black fluid stinking blood from the heart of a patient dead of gangrene of the bowels: a month afterwards the animal was well and vigorous. Another, of three pounds and a quarter, with the blood of an aneurismatic patient, of which twelve grains were used; and the third, of two pound two ounces, with fourteen grains of the same blood, which by its quantity only would probably be fatal: on the twenty-sixth day afterwards the larger rabbit was sprightly and vigorous, the smaller dull and out of condition, with a large wound on the seat of the inoculation.

Dr. Namias considering the reaction in cholera as a beneficial effort of the vital powers to eliminate from the body matters that are incongruous with its tissues, to restore the deranged state of the sanguiferous system, and to renew the exhausted nervous energy, was inclined to believe that the blood of persons dying in this stage would be less injurious than that from persons in the pulseless state; and some experiments that he undertook with Professor Rima seemed to confirm this notion. Twelve grains of the blood of a female, dead after three days' illness with imperfect reaction, were inserted in the usual way into two rabbits, and a third was made to swallow a scruple of the same blood. All three survived, but a great number of spots, produced by the drying of a white glutinous matter, were found in the hutch of the two former. The other appeared to vomit the blood it had swallowed, and for some days after it vomited a similar white substance. Four scruples were injected under the skin at the back of the neck in a little dog. This blood had been taken from a woman dead of cholera in seventy-two hours after incomplete reaction. For the first two days the dog passed along with natural fæces many small white worms; on the third day he vomited milk he had lapped; and he died early on the fourth day. Incipient suppuration was observed under the separated skin; the bladder was quite empty; the heart contained very fluid blood in small quantity; and there was no evident cause of death.

*Bullettino delle Scienze Mediche. September*, 1836.

---

*An Experimental Examination into the Opinions of* Sir Charles Bell *relative to the anatomical and physiological Characters of the Spinal Marrow.* By Henry H. Smith, m.d., resident Physician in the Pennsylvania Hospital.

Believing that the experiments of Sir Charles Bell on this subject demanded a closer examination, particularly as many of them seemed to be based on somewhat supposititious grounds, I repeated a number, and especially those made to prove the connexion of the middle column with the function of respiration. In order to conduct my examination in as impartial a manner as possible, I in each instance pursued the following order: first, to read only the experiment as stated by the author; and, second, to repeat it as nearly as possible in the same way; always, after the first operation, dissecting the portion operated on, so as to make myself sure of its correctness.

. . .

Commencing with his experiments on the anterior and posterior columns of the spinal marrow, I found, after having repeated them several times in various ways, that they all tended to confirm the opinion which he had expressed of their function. Passing over, therefore, the detail of these experiments, (which, as they proved nothing new, might not repay the perusal,) let us proceed to examine the grounds on which he has founded his opinion of the existence of a middle column.

. . . . .

*Experiment* 1*st*. A good-sized kitten was struck lightly on the head, so as to stun it slightly, but without affecting its respiration. The skin was then dissected back, and the muscles removed from the spinous process of the sixth, seventh, and eighth dorsal vertebræ. The points of the scissors being then carefully introduced, the processes were removed, and the canal opened for about two inches. The cord was now raised upon the forceps, and a thin, flat instrument, somewhat similar to a gum lancet, introduced on the right side at the lateral fissure, and carried through to the opposite side. The action of the abdominal muscles, as well as the intercostals, were closely watched by a friend during this operation, but no change in their movements was visible. The instrument, being withdrawn, was again introduced with its flat side to the sacrum, and pushed through so as to separate, for the space of two lines, the anterior from the posterior columns; but without having any effect on the muscles of respiration. By this mode of operating, the anterior column was of course separated from the posterior, but remained otherwise uninjured; while, at the same time, any middle portion must necessarily have been divided. The instrument being removed, the animal continued breathing as readily, apparently, as if unhurt.

*Experiment 2d.* By the same operation, the spinal marrow was exposed at the upper dorsal vertebra. The same instrument was in like manner introduced between the columns here, and allowed to remain some minutes, but without producing any change in the action of the muscles. The animal becoming faint from loss of blood, the instrument was removed, respiration continuing easy. Death was now instantly caused by the insertion of the knife between the occiput and atlas.

*Experiment 3d.* The spinal marrow of another kitten was exposed between the last cervical and first dorsal vertebræ, and immediately divided with the scissors in a transverse direction. This paralyzed the lower extremities, but produced no material change in the action of the abdominal muscles, except that their expansion and contraction was not quite so full as before. The change was, however, very slight; but it was very evident here that the abdominal muscles did not act by themselves, but that they were materially influenced by the diaphragm, as they expanded and contracted at the same instant that it ascended and descended. The cord was afterwards divided at the fifth cervical vertebra, but without any very material change, except that it rendered the action of the diaphragm more plain and completely paralyzed the intercostal muscles.

*Experiment 4th.* The cord was exposed in the spinal canal of another kitten, and divided at the middle dorsal vertebra. In this instance the abdominal muscles were affected in the same manner as in a former experiment, although the effect upon respiration was rather more marked in this instance, as the operation was performed more easily, and the animal was allowed to live for a longer time. At the close of some minutes, respiration still continuing nearly natural, life was destroyed by the division of the cord at the first cervical vertebra.

These experiments were repeated twice on other kittens, and with the same results.

*Experiment 5th.* By a transverse incision on the occiput, the skin of a kitten two months old was divided, and then dissected forward to the crown of the head, and backwards to the sixth cervical vertebra. The spinous processes of the last three cervical vertebræ were now carefully removed with the scissors, and the cord exposed. The roots of the seventh, sixth, and fifth nerves were next cut, when respiration, which before went on naturally, became slightly laboured. The roots of the three next nerves were then also divided, when the action of the diaphragm ceased entirely, and likewise that of the abdominal and intercostal muscles. The animal, however, did not die instantaneously, but continued gasping for near a minute, when death took place; but, previous to this event, motion was observed in the extremities and tail, which was increased on touching the cord. Sensation was not tried, as the time was too short between the division of the roots and the death of the animal; but it no doubt remained, as well as motion.

Had there been in the spinal cord any middle portion especially devoted to respiration, as Sir Charles supposed, the division of it would have been caused by Experiment 1st, as, owing to the manner of operating, its division was ensured, while at the same time the other columns would remain uninjured; thus preventing the action of the muscles supplied by them from being interrupted. According, however, to his theory, this division ought to have arrested the action of the respiratory muscles; whilst, on the contrary, they were not all affected by it! If, again, we admit that an injury to the upper part of the spine does affect respiration, then we must acknowledge that it does so through the medium of the anterior cord and roots; for Experiments 3d and 4th shew that the division of these portions rendered respiration laborious to a slight degree, though it did not stop it entirely. We are, therefore, I think, justified in believing that there is no distinct middle column devoted to respiration; but that, so far as the muscles *are* influenced by nerves below the fifth cervical vertebra, they receive that influence through the anterior roots. Did not these experiments induce us to believe this, the fourth alone would almost do so, as we there see that the division of the upper cervical nerves not only stopped the action of the diaphragm, but also that of the abdominal and intercostal muscles. That the cessation of motion in these muscles was not caused by injury to the anterior column, is apparent from the motion observed in the hind legs after the operation, as well as from the impossibility of such a mishap occurring from the way in which the operation was performed. From these results, I think, we must conclude that the diaphragm is the only proper respiratory muscle, and that the abdominal and intercostal ones are only secondary and mechanical in their action.

*The American Journal of the Medical Sciences. August,* 1837.

